# Net energy reduction and intended slow starch formulation decreased fat deposition in broiler chickens offered conventional and reduced crude protein diets

**DOI:** 10.1016/j.psj.2026.107643

**Published:** 2026-08-18

**Authors:** Damilola U. Kareem, Eunjoo Kim, Shemil P. Macelline, Tahir Mahmood, Yves Mercier, Mehdi Toghyani, Peter H. Selle, Sonia Y. Liu

**Affiliations:** a Poultry Research Foundation, The University of Sydney, Camden NSW 2570, Australia; b School of Life and Environmental Sciences, Faculty of Science, The University of Sydney, Sydney NSW 2006, Australia; c Adisseo France S.A.S., European Labs for Innovation, Science and Expertise (ELISE) 20 Rue Prosper Monnet, Saint Fons, 69190, France; d Sydney School of Veterinary Science, Faculty of Science, The University of Sydney, Sydney NSW 2006, Australia

**Keywords:** Energy metabolism, Net energy, Reduced protein diet, Slowly digestible starch, Starch-protein digestive dynamics

## Abstract

The drawbacks of reduced-crude protein (RCP) diets could include poor growth performance and increased fat deposition. Formulating diets with reduced net energy (NE) densities and slower starch digestion rates could mitigate fat deposition without compromising growth performance. Therefore, this study determined how different NE levels and starch digestion rates influence the performance in broilers offered 205 and 175 g/kg CP grower and 190 and 160 g/kg CP finisher diets. The study comprised a 2 × 2 × 2 factorial arrangement with two NE levels (conventional and 75 kcal/kg reduction), two starch digestion rates (with and without field pea inclusion), and two CP levels (standard and 30 g/kg reduction). Each dietary treatment had 7 replicates with 6 birds per replicate, totaling 336 off-sex male Ross 308 broiler chickens over feeding phases of 11-25 and 25-35 d. Conventional NE during d 11-25 and 25-35 periods were 2350 and 2400 kcal/kg, respectively; while the standard CP were 205 and 190 g/kg, respectively. Overall (d 11 – 35), RCP diets compromised growth performance and increased fat deposition (13.8 versus 11.6 g/kg, *P<* 0.05). Reducing dietary NE significantly reduced relative fat-pad weights by 9.02% (12.1 versus 13.3 g/kg), as did slowly digestible starch (SDS) by 7.58% (12.2 vs 13.2 g/kg). At day 35, the birds’ abdominal fat pad was positively correlated ileal starch: protein disappearance rate ratio (r = 0.610; *P<* 0.001) and breast weight was negatively with ileal starch: protein disappearance rate ratio (r = – 0.485; *P<* 0.001). In conclusion, moderate reductions in dietary NE can be successfully incorporated into reduced-CP diets to reduce fat pad deposition; however, it remains challenging to maintain growth performance in RCP diets.

## Introduction

Energy and protein are important considerations in broiler diet formulation, with starch being the single largest contributor to dietary energy. In practical diets, starch is predominantly derived from cereal grains such as wheat, sorghum, and maize (Liu and Selle, 2015). Reducing dietary protein level inevitably increases the proportion of starch in isoenergetic diets, which may alter the dietary net energy value even when apparent metabolizable energy (AME) is held constant (Kareem et al., 2025). This compositional shift may have resulted in compromised growth performance and increased abdominal fat deposition in broilers. In the present study, reduced crude protein (CP) diets refer to diets formulated with higher concentrations of non-bound amino acids (NBAA) and lower inclusion of intact protein sources to meet the amino acid requirements of animals. Compared with conventional diets, RCP diets typically contain greater proportions of cereal grains and crystalline amino acids, and lower inclusions of soybean meal (SBM) and other protein meals. If not carefully balanced, these diets may also result in reduced dietary electrolyte balance (DEB) and lower supplies of non-essential amino acids, which can potentially compromise bird performance. Interestingly, regardless of whether growth performance is compromised by dietary CP reduction, increased fat pad deposition is consistently observed (Chrystal et al., 2021b). Increased fat deposition in broilers offered RCP diets may be caused by surplus energy intake (Reis et al., 2022), increased dietary starch (Chrystal et al., 2021a; Kareem et al., 2025), or asynchrony in digestion rates (van den Borne et al., 2006).

Macelline et al. (2023) explored the possibility of reducing fat pad deposition in RCP diets by lowering dietary AME density. They reported that reducing dietary CP from 210 to 170 g/kg linearly increased abdominal fat pad weights, whereas a 0.5 MJ/kg or 120 kcal/kg reduction in dietary energy had no effect on abdominal fat pad weights in broilers at 36 days of age. The NE system has been proposed as a more appropriate framework for formulating RCP diets to prevent body fat deposition (Maharjan et al., 2021). It takes into consideration energy losses associated with digestion, absorption, and nutrient metabolism, including the heat increment of protein (Kareem et al., 2025). Consequently, the NE system may provide a more accurate prediction of energy requirements for growth, thereby potentially contributing to leaner broiler production, particularly in the context of RCP diets.

Moreover, Greenhalgh et al. (2022) explored whether capping dietary starch-to-protein ratios could reduce abdominal fat deposition. They reported that relative fat pad weight was not correlated with ileal digestible starch intake *per se* (*P* *>* 0.05), but was positively correlated with dietary starch-to-protein ratios (r = 0.841, *P* *<* 0.05) and ileal digestible starch-to-protein ratios (r = 0.905, *P* *<* 0.05). The duodenum and jejunum are the primary sites of glucose and amino acid absorption in broiler chickens (Riesenfeld et al., 1980), where previous research has shown that approximately 96% of digestible starch and 79% of digestible protein disappearance occurs by the end of the jejunum (Liu et al., 2014). Although it remains unclear, it has been hypothesized that a high simultaneous luminal availability of glucose and amino acids may result in competition at the transporter level, thereby reducing the absorption efficiency of one or both nutrient classes (Liu and Selle, 2015, 2017; Moss et al., 2018; Selle and Liu, 2019; Liu et al., 2021). This potential interaction may be particularly relevant in RCP diets, where intact protein sources, often soybean meal, are partially replaced with NBAA. Hence, including slowly digestible starch may provide more balanced glucose and amino acid absorption and improve nutrient utilization in broilers fed RCP diets (Liu and Selle, 2015; Mahmood et al., 2024).

Overall, the present study aimed to confirm the impact of RCP diets on growth performance and fat pad deposition, and to determine whether reducing dietary NE density and including a source of potentially slowly digestible starch (SDS), through the incorporation of field peas to partially replace SBM and cereal grains, could help reduce fat deposition. It is hypothesized that reducing dietary NE in conjunction with using SDS would help lower fat deposition while maintaining growth performance.

## Materials and methods

### Animal ethics

All procedures in this experiment strictly adhered to the guidelines approved by the Animal Ethics Committee of the University of Sydney (Approval No.: 2024/2527).

### Experimental birds, housing and management

A total of 336 one-day-old off-sex male Ross 308 broiler chicks, with an average weight of 41 g/b were obtained from a commercial hatchery (Goulburn, NSW 2580). Upon arrival, the birds were fed a common starter diet from d 0 to 11 post-hatch. On d 11, the birds were individually identified (wing-tagged), weighed and randomly allocated to eight dietary treatments, each with seven replicate cages, totaling 56 cages (6 birds per cage). Uniformity in average body weight across cages was ensured, with a mean body weight of 396 ± 1.5 g/b. The cages have dimensions of 750 × 750 × 500 mm in length, width and height. Birds had unrestricted access to feed and water in an environmentally controlled facility. A lighting program of 23 h light and 1 h dark was used during the first week and gradually adjusted to 18 h light and 6 h dark from the end of the first week until the end of the experiment (d 35 post-hatch). An initial room temperature of 32 ± 1°C was maintained for the first week, then gradually decreased to 22 ± 1°C by the end of the third week, and maintained at this temperature for the duration of the feeding trial.

### Experimental design

This study was laid in a 2 × 2 × 2 factorial arrangement comprising two dietary NE levels (conventional or low), two starch sources (rapidly or slowly digestible), and two dietary CP levels (standard or 30 g/kg reduction). The outline of the dietary treatments, dietary treatment description and feeding regimes are presented in Table 1.

**Table 1:** Description of the dietary treatments and feeding regimes.

	Experiment Outline			Feeding regimes					
				Grower (d 11 - 25)			Finisher (d 25 - 35)		
Diet	Net Energy Level^1^	Starch source	Dietary CP Level^2^	NE, kcal/kg	Starch source	CP, g/kg	NE, kcal/kg	Starch source	CP, g/kg
1	Conventional	RDS^4^	Standard	2350	RDS	206	2400	RDS	190
2	Conventional	RDS	Reduced^6^	2350	RDS	175	2400	RDS	160
3	Conventional	SDS^5^	Standard	2350	SDS	205	2400	SDS	190
4	Conventional	SDS	Reduced	2350	SDS	175	2400	SDS	160
5	Low^3^	RDS	Standard	2275	RDS	206	2325	RDS	190
6	Low	RDS	Reduced	2275	RDS	175	2325	RDS	160
7	Low	SDS	Standard	2275	SDS	205	2325	SDS	190
8	Low	SDS	Reduced	2275	SDS	175	2325	SDS	161

1 Net Energy calculated using the equation 2 of Noblet et al. (2024) from recommended AME value of Ross 308.

2 Dietary crude protein level.

3 Net energy reduction by 75 kcal/kg.

4 RDS: Rapidly digestible starch (Maize).

5 SDS: Slowly digestible starch (Maize diluted with field peas).

6 Dietary protein reduction by 30 g/kg.

### Experimental diets

Prior to diet formulation, corn, wheat, soybean meal, meat and bone meal, peas, millrun and solvent canola meal were analyzed by near-infrared spectroscopy to predict proximate analysis, digestible amino acid concentrations, and apparent metabolizable energy (AME) using Near-Infrared (NIR) spectroscopy. The birds were initially provided with a common starter diet from 0 to 11 d post-hatch. Experimental diets were fed during the grower (d 11 – 25) and finisher (d 25 – 35) phases. Starter and four of the experimental diets (grower and finisher) were formulated according to Ross 308 nutrient specification (Aviagen, 2022), while four experimental diets had low dietary CP (30 g/kg reduction from the specification). Titanium dioxide (TiO_2_) was used as an indigestible marker in the finisher diets to calculate apparent nutrient digestibility. Rapidly digestible starch (RDS) was provided by maize, whereas slow starch digestion was achieved through the inclusion of field peas. The standard CP diets were formulated to contain 205 and 190 g/kg CP in the grower and finisher phases, respectively, whereas the reduced CP diets contained 175 and 160 g/kg CP. The experimental diets were formulated to contain a fixed inclusion rate of wheat, with varying maize contents. Experimental diets were steam-pelleted at 80°C. The pellet mill was equipped with a die ring with 4.0 mm holes and 38 mm thickness. Crumbled diets were fed to the birds from d 0 – 15 while pellets were fed from 15 d post-hatch to the end of the experiment (35 d post-hatch). The dietary NE values of conventional NE diets were calculated from recommended AME value of Ross 308 nutrient specification (Aviagen, 2022), while the low NE diets were lowered by 75 kcal/kg. The NE was calculated using equation 4 of Noblet et al. (2024), considering the dietary AME (MJ/kg), CP (%), ether extract (%) and neutral detergent fiber (NDF; %) as shown below:

$$NE_{\mathrm{diet}}\left(\mathrm{MJ}/\mathrm{kg}\right)=0.804\times \text{AME}-0.026\times \text{CP}+0.015\times \text{ether}\;\text{extract}-0.018\times \text{NDF}$$

The NE is reported in kcal/kg using the conversion factor 239 kcal = 1 MJ. The compositions of the experimental diets at the grower (d 11 – 25) and finisher (d 25 – 35) phases are shown in Table 2, Table 3, respectively, while the analyzed total amino acid profile of the experimental diets at the finisher phase is presented in Table 4.

**Table 2:** Ingredient composition of experimental diets at the grower phase (d 11-25)^1^.

			Starter		Grower (d 11-25)							
	Ingredient price/tonne (AU$)				Conventional NE				Low NE (−75kcal)			
					RD Starch		SD Starch		RD Starch		SD Starch	
Items (g/kg)					SCP	RCP	SCP	RCP	SCP	RCP	SCP	RCP
Maize (80 g/kg CP)	400		403		442	513	314	406	441	464	334	357
Wheat (115 g/kg CP)	400		200		200	200	200	200	200	200	200	200
Soybean Meal (470 g/kg CP)	750		301		263	120	184	52	259	106	181	38
Canola Meal Solvent (365 g/kg CP)	450		50		50	50	50	50	50	50	50	50
Peas	400		-		-	-	200	200	-	-	200	200
L-Lysine HCl	2500		3.50		1.86	6.12	1.36	5.35	1.91	6.39	1.43	5.61
DL-Methionine	4500		3.60		2.45	3.69	3.04	4.19	2.44	3.76	3.02	4.26
L-Threonine	2500		1.85		1.08	2.98	1.37	3.13	1.09	3.11	1.37	3.26
L-Arginine	6500		0.56		-	3.41	-	2.32	-	3.53	-	2.44
L-Glycine	6000		-		0.18	3.64	0.54	3.78	0.15	3.76	0.54	3.90
L-Tryptophan	13000		-		-	0.12	-	0.31	-	0.10	-	0.30
L-Isoleucine	7500		0.55		-	2.34	0.32	2.56	-	2.52	0.34	2.75
L-Leucine	5000		-		-	0.32	-	1.26	-	0.81	-	1.74
L-Valine	4500		0.74		-	2.35	0.44	2.63	-	2.49	0.44	2.77
Millrun (150 g/kg CP)	400		-		-	57	-	33	17	120	-	96
Canola Oil	1950		6.50		16.56	5.00	22.09	5.00	5.00	5.00	5.56	5.00
Limestone	119		13.35		11.21	11.44	11.15	11.36	11.22	11.41	11.18	11.35
Mono Dicalcium Phosphate	1650		8.07		4.46	6.44	4.23	6.10	4.42	6.43	4.22	6.10
Potassium Carbonate	2400		-		-	1.87	-	1.61	-	1.53	-	1.27
Sodium Bicarbonate	1200		2.10		1.84	4.31	1.32	3.62	1.89	4.54	1.36	3.85
Sodium Chloride	239		2.25		2.47	0.83	2.51	0.99	2.44	0.68	2.48	0.84
Vit/Min Premix	9540		2.00		2.00	2.00	2.00	2.00	2.00	2.00	2.00	2.00
Choline Chloride (600 g/kg purity)	2500		0.95		0.94	1.64	1.27	1.94	0.93	1.64	1.27	1.94
Rovabio® Adv PHY (1000 FTU)^4^	8500		0.10		0.10	0.10	0.10	0.10	0.10	0.10	0.10	0.10
Total			1000		1000	1000	1000	1000	1000	1000	1000	1000
Total NBAA			10.03		5.16	23.62	6.76	24.35	5.17	25.06	6.82	25.80
PDI (%)			90.9		83.8	93.7	91.6	95.0	92.6	96.4	95.1	97.6
Price (AU$/T)			613		587	628	565	598	572	630	544	600
												
**Calculated nutrient composition (g/kg unless otherwise stated)**												
DM			902		901	899	902	900	899	897	900	898
NE (kcal/kg)			2250		2350	2350	2350	2350	2275	2275	2275	2275
AME (kcal/kg)			2988		3089	3084	3086	3079	3009	3006	3005	3002
Crude Protein			225		206	175	205	175	206	175	205	175
Starch			391		416	473	421	487	419	453	434	467
Crude fat			30.32		41.45	33.21	44.95	31.37	30.46	33.14	29.32	31.30
Crude fiber			26.84		26.13	27.63	32.33	32.29	27.52	32.15	32.52	36.81
Neutral detergent fiber			103.88		104.16	122.47	103.71	114.71	110.80	142.98	105.34	135.21
Ash			54.76		48.34	46.04	46.67	44.54	48.32	45.08	46.78	43.60
Calcium			9.5		8.00	8. 01	8.00	8.00	8.00	8.00	8.00	8.00
Available Phosphorus			4.7		4.00	4.00	4.00	4.00	4.00	4.00	4.00	4.00
Sodium			2.00		2.00	2.00	2.00	2.00	2.00	2.00	2.00	2.00
Chloride			2.60		2.40	2.34	2.40	2.34	2.40	2.34	2.40	2.34
Potassium			8.29		7.72	7.00	7.85	7.00	7.81	7.00	7.85	7.00
DEB (meq/kg)^2^			226		217	200	220	200	219	200	220	200
Dig. Lys			13.2		11.00	11.00	11.00	11.00	11.00	11.00	11.00	11.00
Dig. Met			6.68		5.40	6.01	5.64	6.21	5.39	6.04	5.63	6.24
Dig. M+C			10.03		8.58	8.58	8.58	8.58	8.58	8.58	8.58	8.58
Dig. Thr			8.98		7.70	7.70	7.70	7.70	7.70	7.70	7.70	7.70
Dig. Ile			8.98		7.78	7.70	7.70	7.70	7.76	7.70	7.70	7.70
Dig. Leu			16.2		15.33	12.10	14.04	12.10	15.30	12.10	14.11	12.10
Dig. Trp			2.54		2.34	1.76	2.12	1.76	2.35	1.76	2.11	1.76
Dig. Arg			13.99		12.35	11.88	13.22	11.88	12.35	11.88	13.17	11.88
Dig. His			5.23		4.89	3.66	4.66	3.51	4.90	3.59	4.66	3.45
Dig. Val			10.16		8.80	8.80	8.80	8.80	8.80	8.80	8.80	8.80
Dig. Phe			9.28		8.64	6.25	8.22	6.01	8.63	6.06	8.23	5.83
Dig. Pro			12.04		11.55	9.60	10.49	8.72	11.58	9.42	10.55	8.54
Dig. Ala			8.86		8.42	6.67	7.82	6.24	8.43	6.48	7.87	6.05
Dig. Asp			17.49		16.00	10.46	15.95	10.82	15.93	10.02	15.90	10.38
Dig. Glu			37.75		35.52	27.39	34.19	26.61	35.57	26.97	34.22	26.19
Dig. Gly			7.45		7.12	8.80	7.44	8.99	7.12	8.94	7.43	9.13
Dig. Ser			9.7		9.07	6.72	8.63	6.45	9.07	6.53	8.63	6.26
Dig. Gly_equi_^3^			14.38		13.60	13.60	13.60	13.60	13.60	13.60	13.60	13.60
**Analyzed nutrient values (g/kg unless otherwise stated)**												
Gross energy (kcal/kg)			4414		4470	4393	4475	4386	4397	4404	4406	4389
Crude protein			237		200	182	214	180	206	179	193	183
Crude fat			28		37	31	40	29	28	31	28	28
Starch			384		412	450	401	456	394	443	414	425
Amylose			140		83	134	125	133	124	131	124	128
Amylopectin			244		329	316	276	323	270	312	290	297
Amylose:amylopectin			0.57		0.25	0.42	0.45	0.41	0.46	0.42	0.43	0.43

1 PDI: Pellet Durability Index (means of 4 replicate measurements), NE: Net Energy, RD: Rapidly digestible, SD: Slowly digestible, SCP: Standard crude protein, RCP: Reduced crude protein, NBAA: Non-bound amino acid.

2 DEB: dietary electrolyte balance = K^+^ + Na^+^ - Cl^-^.

3 Glycine equivalent = glycine concentration (g/kg) + [serine concentration (g/kg) * 0.7143].

4 Contains 6-phytase, β-glucanase, arabinofuranosidase and xylanase at the activities ≥10,000 FTU/g, ≥8,600 VU/g, ≥ 46,000 VU/g and ≥12,500 VU/g, respectively (Rovabio® Advance PHY, Adisseo S.A.S., Antony, France).

**Table 3:** Ingredient composition of experimental diets at the finisher phase (d 25-35)^1^.

	Ingredient price/tonne (AU$)		Conventional NE					Low NE (−75 kcal)			
			RD Starch		SD Starch			RD Starch		SD Starch	
Items (g/kg)			SCP	RCP	SCP	RCP		SCP	RCP	SCP	RCP
Maize (80 g/kg CP)	400		471	562	341	455		490	513	361	404
Wheat (115 g/kg CP)	400		200	200	200	200		200	200	200	200
Soybean Meal (470 g/kg CP)	750		216	74	140	7		213	60	136	-
Canola Meal Solvent (365 g/kg CP)	450		60	60	60	60		60	60	60	60
Peas	400		-	-	200	200		-	-	200	200
L-Lysine HCl	2500		2.14	6.40	1.56	5.62		2.16	6.66	1.62	5.66
DL-Methionine	4500		2.37	3.60	2.93	4.09		2.34	3.67	2.92	4.10
L-Threonine	2500		1.18	3.07	1.43	3.22		1.17	3.20	1.44	3.25
L-Arginine	6500		-	3.89	-	2.79		-	4.01	-	2.72
L-Glycine	6000		-	3.40	0.22	3.55		-	3.52	0.22	3.48
L-Tryptophan	13000		-	0.23	0.00	0.43		-	0.21	0.00	0.37
L-Isoleucine	7500		0.19	2.60	0.55	2.82		0.19	2.78	0.57	2.88
L-Leucine	5000		-	0.38	-	1.32		-	0.87	-	1.60
L-Valine	4500		0.16	2.51	0.56	2.79		0.15	2.65	0.56	2.80
Millrun (150 g/kg CP)	400		-	41	-	16		-	103	-	74
Canola Oil	1950		21.26	5.00	27.31	5.00		5.00	5.00	10.78	5.00
Limestone	119		11.07	11.29	11.00	11.23		11.10	11.28	11.03	11.21
Monocalcium Phosphate	1650		2.03	4.03	1.76	3.70		2.00	4.03	1.75	3.59
Potassium Carbonate	2400		-	2.25	-	1.99		-	1.91	-	1.49
Sodium Bicarbonate	1200		2.20	4.65	1.65	3.96		2.22	4.88	1.68	4.06
Sodium Chloride	239		2.33	0.61	2.29	0.76		2.21	0.45	2.26	0.69
Vit/Min Premix	9540		2.00	2.00	2.00	2.00		2.00	2.00	2.00	2.00
Choline Chloride (600 g/kg purity)	2500		0.95	1.66	1.27	1.96		0.94	1.67	1.27	1.93
Rovabio® Adv PHY (1000 FTU)^4^	8500		0.10	0.10	0.10	0.10		0.10	0.10	0.10	0.10
TiO_2_			5.00	5.00	5.00	5.00		5.00	5.00	5.00	5.00
Total			1000	1000	1000	1000		1000	1000	1000	1000
Total NBAA			5.57	24.67	6.91	25.39		5.54	26.11	6.97	25.62
PDI (%)			84.6	90.0	88.2	94.7		91.0	93.7	92.5	94.8
Price (AU$/T)			577	620	556	590		557	621	535	587
											
**Calculated nutrient composition (g/kg unless otherwise stated)**											
DM			901	899	902	900		899	897	901	898
NE (kcal/kg)			2400	2400	2400	2400		2325	2325	2325	2325
AME (kcal/kg)			3136	3130	3134	3126		3055	3052	3052	3048
Crude Protein			190	160	190	160		190.4	160	190	161.41
Starch			435	501	438	515		447	481	451	493
Crude fat			47.1	34.43	51.03	32.59		31.7	34.36	35.41	32.39
Crude fiber			26.01	26.3	32.26	30.95		26.23	30.81	32.46	35.24
Neutral detergent fiber			105.5	118.59	105.02	110.81		107.11	139.08	106.65	129.73
Ash			44.9	43.09	43.3	41.61		45.05	42.15	43.41	40.77
Calcium			7.50	7.50	7.50	7.50		7.50	7.50	7.50	7.50
Available Phosphorus			3.50	3.50	3.50	3.50		3.50	3.50	3.50	3.50
Sodium			2.00	2.00	2.00	2.00		2.00	2.00	2.00	2.00
Chloride			2.30	2.24	2.3	2.24		2.30	2.24	2.30	2.24
Potassium			7.09	6.5	7.25	6.5		7.11	6.5	7.25	6.5
DEB (meq/kg)^2^			203	190	208	190		204	190	208	190
Dig. Lys			10.2	10.2	10.2	10.2		10.2	10.2	10.2	10.2
Dig. Met			5.15	5.76	5.38	5.96		5.13	5.79	5.37	5.95
Dig. M+C			8.16	8.16	8.16	8.16		8.16	8.16	8.16	8.16
Dig. Thr			7.24	7.24	7.24	7.24		7.24	7.24	7.24	7.24
Dig. Ile			7.24	7.24	7.24	7.24		7.24	7.24	7.24	7.24
Dig. Leu			14.28	11.22	13.05	11.22		14.38	11.22	13.12	11.22
Dig. Trp			2.12	1.63	1.91	1.63		2.11	1.63	1.9	1.63
Dig. Arg			11.14	11.12	12.08	11.12		11.13	11.12	12.04	11.12
Dig. His			4.51	3.29	4.3	3.14		4.53	3.22	4.31	3.14
Dig. Val			8.26	8.26	8.26	8.26		8.26	8.26	8.26	8.26
Dig. Phe			7.9	5.53	7.53	5.3		7.92	5.34	7.53	5.24
Dig. Pro			10.96	9.05	9.93	8.17		11.04	8.87	10	8.1
Dig. Ala			7.9	6.19	7.33	5.76		7.97	6	7.38	5.67
Dig. Asp			14.27	8.77	14.32	9.13		14.26	8.33	14.27	8.99
Dig. Glu			33.06	24.93	31.87	24.15		33.16	24.52	31.9	24.17
Dig. Gly			6.41	8	6.63	8.19		6.42	8.14	6.62	8.24
Dig. Ser			8.35	6.01	7.94	5.75		8.38	5.83	7.95	5.69
Dig. Gly_equi_^3^			12.4	12.3	12.3	12.3		12.4	12.3	12.3	12.3
**Analyzed nutrient values (g/kg unless otherwise stated)**											
Gross energy (kcal/kg)			3917	3867	3976	3870		3917	3868	3890	3861
Crude protein			199	166	187	157		187	164	191	167
Crude fat			35	36	49	33		33	31	32	30
Starch			427	462	408	469		434	439	426	467
Amylose			126	138	170	190		139	108	160	138
Amylopectin			301	324	238	279		295	331	266	329
Amylose:amylopectin			0.42	0.43	0.71	0.68		0.47	0.32	0.60	0.42

1 PDI: Pellet Durability Index (means of 4 replicate measurements), NE: Net Energy, RD: Rapidly digestible, SD: Slowly digestible, SCP: Standard crude protein, RCP: Reduced crude protein, NBAA: Non-bound amino acid.

2 DEB: dietary electrolyte balance = K^+^ + Na^+^ - Cl^-^.

3 Glycine equivalent = glycine concentration (g/kg) + [serine concentration (g/kg) * 0.7143].

4 Contains 6-phytase, β-glucanase, arabinofuranosidase and xylanase at the activities ≥10,000 FTU/g, ≥8,600 VU/g, ≥ 46,000 VU/g and ≥12,500 VU/g, respectively (Rovabio® Advance PHY, Adisseo S.A.S., Antony, France).

**Table 4:** Analyzed total amino acid profile of experimental diets at the finisher phase (d 25-35).

	Conventional NE				Low NE (−75 kcal)			
	RD Starch		SD Starch		RD Starch		SD Starch	
Total amino acids (g/kg)	SCP	RCP	SCP	RCP	SCP	RCP	SCP	RCP
Histidine	23.6	21.5	22.8	20.9	23.6	21.1	23.1	20.6
Serine	63.9	55.5	60.8	53.9	62.8	53.6	61.3	53.3
Arginine	45.7	51.2	49.4	54.6	45.9	52.6	50.4	53.4
Glycine	80.5	100.5	78.0	103.8	78.9	106.4	81.0	114.7
Aspartic acid	85.4	67.5	89.4	68.2	88.1	62.8	84.7	67.9
Glutamic acid	174.2	160.8	178.2	156.7	175.8	161.0	173.9	155.8
Threonine	50.0	54.6	50.5	56.6	49.4	54.8	51.6	55.3
Alanine	76.2	72.9	72.0	65.4	75.8	69.8	71.3	65.1
Proline	81.1	77.6	73.3	71.0	79.0	77.6	75.2	69.6
Lysine	51.0	58.0	54.3	63.0	53.3	59.5	54.0	61.8
Tyrosine	17.1	17.3	18.5	15.5	16.7	16.4	18.5	15.8
Methionine	20.0	29.6	25.9	30.4	20.2	29.7	25.8	32.0
Valine	57.3	64.1	57.8	69.2	57.3	66.5	59.2	67.0
Isoleucine	45.5	48.7	46.4	52.8	45.1	49.6	46.7	51.3
Leucine	89.0	85.9	84.6	84.4	89.0	85.2	84.9	83.8
Phenylalanine	39.6	34.4	37.9	33.6	39.3	33.4	38.4	32.5

NE: Net Energy, RD: Rapidly digestible, SD: Slowly digestible, SCP: Standard crude protein, RCP: Reduced crude protein.

### Data and sample collection, chemical analyses, and calculations

Diets were analyzed for pellet durability index (PDI) using the NHP 200 New Holman Automatic Pellet Tester (TekPro Ltd, Norfolk, UK).

Birds were weighed on a cage basis on d 11, 25, and 35 to determine body weights (BW) and calculate body weight gain (BWG). Feed intake (FI) was recorded over the same intervals and used to calculate the feed conversion ratio (FCR). Mortality was recorded daily, and FCR was corrected for mortality. Costs per kg of diets were calculated from the market prices of raw materials in Australia at the time of feed formulation (September 2024), exclusive of feed milling expenses. Overall (d 0 – 35) feed cost per kg of live body weight was calculated using diet prices, FI, and FCR, with a fixed FI of 348 g/b for the common starter. Considering the calculated AU$ 612.55 per tonne for the common starter diet (Table 2), the estimated feed cost at the starter phase was AU$ 0.21 per bird. Feed cost/kg of live BW (AU$) of the birds was calculated according to the equation below:

$$Feed\;cost/kg\;BW\;=\;\frac{Feed\;intake\;\times feed\;cost/kg\;of\;diet}{Body\;weight_{d35}}$$

Average BW of the birds at d 0 was 41 g, and the estimated age to 2.5 kg was considered from d 0 – 35 using the equation below:

$$\mathrm{Estimated}\;\mathrm{age}\;\mathrm{to}\;2.5\;\mathrm{kg}\;=\;\frac{2500\;g}{\mathrm{Average}\;\mathrm{Daily}\;\mathrm{weight}\;\mathrm{gain},\;g}$$

Total excreta output and feed intake were recorded on a cage basis from 27 to 29 d post-hatch to determine AME, AME-to-gross energy (GE) ratios (AME:GE), nitrogen (N) retention, and N-corrected AME (AMEn). Dry matter (DM) content of excreta was measured by drying samples in a forced-air oven at 80°C for 24 h (Chrystal et al., 2021a). The GE of excreta and diets was determined using a Parr 1281 adiabatic bomb calorimeter (Parr Instruments Co., Moline, IL, USA). AME on a DM basis was calculated according to the following equation:

$$\text{AM}E_{\text{diet}},kcal/kg=\frac{\left(\text{Feed}\;\text{intake},\;kg\;\times GE_{\text{diet}},\;kcal/kg\right)-\;\left(\text{Excreta}\;\text{output},\;\text{kg}\;\times GE_{\text{excreta}},\;kcal/kg\right)}{\text{Feed}\;\text{intake},\;kg}$$

The N contents of diets and excreta were determined using the Dumas combustion method (Leco Corporation, St Joseph, MI), and N retention was calculated using the following equation:

$$N\;\text{Retention},\;\%=\frac{\left(\text{Feed}\;\text{intake},\;kg\;\times N_{\text{diet}},\;\%\right)-\;\left(\text{Excreta}\;\text{output},\;kg\times N_{\text{excreta}},\;\%\right)}{\left(\text{Feed}\;\text{intake},\;kg\;\times N_{\text{diet}},\;\%\right)}$$

The AMEn values was calculated by correcting N retention to zero (Hill and Anderson, 1958) using the correction factor of 8.73 kcal/g N retained in the body proposed by Titus et al. (1959) according to the equation below:

$$\text{AME}n_{\text{diet}},kcal/kg=AME\;-\left(N\;retained,\;gDM\;\times \;8.73\;kcal/g\right)$$

At 35 d post-hatch, all birds were individually weighed and euthanized by intravenous injection of sodium pentobarbitone for digesta collection and carcass yield. The distal jejunum was determined by the mid-point between the end of the duodenal loop and Meckel’s diverticulum, and the distal ileum by the mid-point between Meckel’s diverticulum and the ileo-caecal junction. Digesta from the distal jejunum and distal ileum was gently expressed and pooled per cage. The abdominal fat pads were removed, weighed to calculate relative weight to the body weight. *Pectoralis major* (breast), *Pectoralis minor* (tenderloins), and thighs (leg quarters) were dissected and weighed to calculate relative weight to the body weights. Both absolute (g/b) and relative (g/kg) weights were reported.

Digesta samples were freeze-dried using VirTis BenchTop Pro 8 L XC freeze-dryer (SP Scientific, Warminster, USA). Feed and freeze-dried digesta samples were ground (Foss CT 293 Cyclotec™, Denmark) to pass through a 0.5 mm screen. TiO_2_ concentration in feed and digesta samples was determined using the procedure described by Short et al. (1996). The total starch content was analyzed using Megazyme Total Starch Assay kit (Megazyme, Wicklow, Ireland, UK) as described in McCleary et al. (1997). The amylose in the diets was analyzed using the Megazyme Amylose/Amylopectin Assay kit (Megazyme,Wicklow, Ireland, UK) as described in the Megazyme (2024) instruction manual. The amylopectin proportion was calculated by subtracting the amylose (g/kg) from 1000. Both amylose and amylopectin were then estimated as proportions of total starch (g/kg), and the amylose:amylopectin ratio was calculated using these values. The N content of diets and digesta was determined using the Dumas combustion method (Leco Corporation, St Joseph, MI). Analyzed N values were then converted to crude protein using a correction factor of 6.25. The apparent digestibility coefficients for starch and crude protein were calculated from the following equation:

$$\text{Digestibility}\;\text{coefficient}=\frac{{\left(\text{Nutrient}/{\text{TiO}}_{2}\right)}_{\text{diet}}-\;{\left(\text{Nutrient}/{\text{TiO}}_{2}\right)}_{\text{digesta}}}{{\left(\text{Nutrient}/{\text{TiO}}_{2}\right)}_{\text{diet}}}$$

Starch and protein disappearance rates (g/d) were calculated using the following equation:

$$\begin{array}{ccc} & & \text{Nutrient}\;\text{disappearance}\;\text{rate},g/b/d=\;\text{Feed}\;\text{Intake},\;\mathrm{kg}/b/d\\ & & \times \;\text{Dietary}\;\text{nutrient},\;g/\mathrm{kg}\;\times \;\mathrm{apparent}\;\mathrm{digestibility}\;\mathrm{coefficient} \end{array}$$

Where FI is the feed intake (g/b/d) of the birds during the excreta collection period (d 27 – 29).

Ratios of starch to protein disappearance rates (S:P) in the distal jejunum and ileum were calculated as this eliminates the confounding influence of feed intake.

### Statistical analysis

Data were checked for normality (Shapiro-Wilk and Anderson-Darling tests) and homogeneity of variances (Levene’s test) and then subjected to statistical analysis using 3-way ANOVA of the GLM procedure in JMP Student Edition 19.0.5 (SAS Institute Inc., JMP Software, Cary, NC) to assess the main effects of NE, CP, starch source, and their interactions. Each cage was considered an experimental unit (*n* = 56) and the values presented in the tables are means with pooled standard error of mean (SEM). If a significant effect of treatment was detected, differences between the means were separated using Tukey HSD test. Significance was assessed at a 5% probability level. Relationships between nutrient utilization and some selected parameters were assessed using Pearson Product–Moment Correlation, with some represented with linear plots using JMP Student Edition 19.0.5.

## Results

### Pellet durability index

The PDI of the experimental diets at the grower (d 11-25) and finisher (d 25 – 35) phases are presented in Table 5. A significant three-way interaction was observed for the PDI at the grower phase (*P* *<* 0.001). Dietary CP reduction and low NE consistently increased PDI when SDS was utilized. At the finisher phase, NE interacted with starch and CP (*P* *<* 0.001) to alter dietary PDI. Both SDS and RCP diets led to higher PDI at both conventional and low NE levels.

**Table 5:** Pellet durability index (PDI, %) of the experimental diets at the grower (d 11-25) and finisher (d 25 – 35) phases.

NE^1^	Starch	CP^2^	Grower	Finisher
***NE × Starch × CP***				
Conventional	RDS^3^	Standard	83.8^f^	84.6
Conventional	RDS	Reduced	93.7^cd^	90.0
Conventional	SDS^4^	Standard	91.6^e^	88.2
Conventional	SDS	Reduced	95.0^bc^	94.7
Low	RDS	Standard	92.6^de^	91.0
Low	RDS	Reduced	96.4^ab^	93.7
Low	SDS	Standard	95.1^bc^	92.5
Low	SDS	Reduced	97.6^a^	94.8
*SEM*			0.33	0.26
***NE × Starch***				
Conventional	RDS		88.7	87.3^d^
Conventional	SDS		93.3	91.4^c^
Low	RDS		94.5	92.3^b^
Low	SDS		96.4	93.7^a^
***NE × CP***				
Conventional		Standard	87.7	86.4^d^
Conventional		Reduced	94.4	92.3^b^
Low		Standard	93.9	91.8^c^
Low		Reduced	97.0	94.2^a^
***Starch × CP***				
	RDS	Standard	88.2	87.8
	RDS	Reduced	95.0	91.8
	SDS	Standard	93.4	90.4
	SDS	Reduced	96.3	94.7
***Main Effects***				
***NE***				
Conventional			91.0	89.4
Low			95.4	93.0
***Starch***				
RDS			91.6	89.8
SDS			94.8	92.6
***CP***				
Standard			90.8	89.1
Reduced			95.7	93.3
***P-Values***				
NE × Starch × CP			<0.001	0.064
NE × Starch			<0.001	<0.001
NE × CP			<0.001	<0.001
Starch × CP			<0.001	0.317
NE			<0.001	<0.001
Starch			<0.001	<0.001
CP			<0.001	<0.001

^a-e^Means with different superscripts across the same column are significantly (*P<* 0.05) different.

SEM: Standard Error of the Mean.

1 Net Energy.

2 Crude Protein.

3 Rapidly Digestible Starch.

4 SlowlyDigestible Starch.

### Growth Performance, Feed Cost and estimated Age to 2.5 kg

During the grower phase (d 11 – 25; Table 6), as a main effect, birds offered RCP diets had lower BW, BWG, FI and higher FCR. No treatment interactions were observed for growth performance parameters and reducing dietary NE reduced FCR (1.531 vs 1.515) while including field peas in the diets also led to decreased FCR (1.532 vs 1.514). During the finisher phase (d 25 – 35; Table 7), birds offered RCP diets presented lower BW (2756 vs. 2883 g/b, *P* *<* 0.001), BWG (1169 vs. 1234 g/b, *P* *<* 0.001), and FI (1925 vs. 2000 g/b, *P* *<* 0.001) and higher FCR (1.621 vs 1.649) compared to standard CP diets regardless of dietary NE and starch source. Overall (d 11 – 35; Table 8), birds offered RCP diets showed significantly lower BWG (2360 vs. 2487 g/b, *P* *<* 0.001), FI (3770 vs. 3873 g/b, *P* *<* 0.001) and higher FCR (1.598 vs. 1.558 g/b, *P* *<* 0.001) compared to the birds offered standard CP diets. There were no treatment interactions for any growth performance metrics over 25-35 and 11-35 days post-hatch.

**Table 6:** Growth performance of broilers fed diets of varying net energy level, starch source and crude protein level at the grower phase (d 11-25)^1^.

NE^2^	Starch	CP^3^	IBW^4^	BW^5^	BWG^6^	FI^7^	FCR^8^, g/g
***NE × Starch × CP***			———————g/b———————				
Conventional	RDS^9^	Standard	396	1614	1218	1859	1.527
Conventional	RDS	Reduced	398	1584	1186	1851	1.561
Conventional	SDS^10^	Standard	395	1658	1263	1873	1.483
Conventional	SDS	Reduced	394	1590	1193	1850	1.552
Low	RDS	Standard	397	1654	1257	1876	1.492
Low	RDS	Reduced	396	1590	1194	1848	1.548
Low	SDS	Standard	396	1667	1271	1885	1.483
Low	SDS	Reduced	396	1586	1191	1830	1.537
*SEM*			1.1	12.1	12.0	16.4	0.0084
***NE × Starch***							
Conventional	RDS		397	1599	1202	1855	1.544
Conventional	SDS		396	1624	1228	1861	1.517
Low	RDS		397	1622	1226	1862	1.520
Low	SDS		396	1626	1231	1857	1.510
***NE × CP***							
Conventional		Standard	395	1636	1240	1866	1.505
Conventional		Reduced	397	1587	1189	1851	1.556
Low		Standard	397	1660	1264	1880	1.488
Low		Reduced	396	1588	1192	1839	1.545
***Starch × CP***							
	RDS	Standard	397	1634	1237	1868	1.510
	RDS	Reduced	397	1587	1190	1849	1.555
	SDS	Standard	395	1662	1267	1879	1.483
	SDS	Reduced	397	1588	1192	1840	1.544
							
***Main Effects***							
***NE***							
Conventional			396	1611	1215	1858	1.531^a^
Low			396	1624	1228	1860	1.515^b^
***Starch***							
RDS			397	1610	1214	1859	1.532^a^
SDS			396	1625	1229	1860	1.514^b^
***CP***							
Standard			396	1648^a^	1252^a^	1873^a^	1.497^b^
Reduced			397	1587^b^	1191^b^	1845^b^	1.549^a^
***P-Values***							
NE × Starch × CP			0.724	0.521	0.528	0.789	0.130
NE × Starch			0.850	0.237	0.210	0.618	0.169
NE × CP			0.058	0.178	0.238	0.272	0.763
Starch × CP			0.381	0.126	0.110	0.375	0.177
NE			0.786	0.142	0.127	0.921	0.012
Starch			0.230	0.093	0.070	0.932	0.003
CP			0.363	<0.001	<0.001	0.018	<0.001

^ab^Means with different superscripts across the same column are significantly different (*P<* 0.05).

SEM: Standard Error of the Mean.

1 No mortality was recorded at this phase.

2 Net Energy.

3 Crude Protein.

4 Initial Body Weight.

5 Body Weight.

6 Body Weight Gain.

7 Feed Intake.

8 Feed Conversion Ratio.

9 Rapidly Digestible Starch.

10 Slowly Digestible Starch.

**Table 7:** Growth performance of broilers fed diets of varying net energy level, starch source and crude protein level at the finisher phase (d 25-35)^1^.

NE^2^	Starch	CP^3^	BW^4^	BWG^5^	FI^6^	FCR^7^, g/g
***NE × Starch × CP***			——————-g/b—————–			
Conventional	RDS^8^	Standard	2830	1216	1962	1.614
Conventional	RDS	Reduced	2785	1201	1931	1.609
Conventional	SDS^9^	Standard	2888	1230	2002	1.628
Conventional	SDS	Reduced	2738	1148	1904	1.658
Low	RDS	Standard	2910	1256	2048	1.631
Low	RDS	Reduced	2761	1171	1935	1.654
Low	SDS	Standard	2902	1236	1987	1.609
Low	SDS	Reduced	2742	1155	1932	1.673
*SEM*			21.6	15.8	22.0	0.0196
***NE × Starch***						
Conventional	RDS		2807	1209	1947	1.612
Conventional	SDS		2813	1189	1953	1.643
Low	RDS		2836	1214	1991	1.643
Low	SDS		2822	1196	1960	1.641
***NE × CP***						
Conventional		Standard	2859	1223	1982	1.621
Conventional		Reduced	2762	1174	1917	1.634
Low		Standard	2906	1246	2018	1.620
Low		Reduced	2751	1163	1933	1.664
***Starch × CP***						
	RDS	Standard	2870	1236	2005	1.623
	RDS	Reduced	2773	1186	1933	1.632
	SDS	Standard	2895	1233	1995	1.619
	SDS	Reduced	2740	1152	1918	1.666
***Main Effects***						
***NE***						
Conventional			2810	1199	1950	1.627
Low			2829	1205	1976	1.642
***Starch***						
RDS			2822	1211	1969	1.627
SDS			2818	1192	1956	1.642
***CP***						
Standard			2883^a^	1234^a^	2000^a^	1.621^b^
Reduced			2756^b^	1169^b^	1925^b^	1.649^a^
***P-Values***						
NE × Starch × CP			0.132	0.118	0.051	0.906
NE × Starch			0.529	0.957	0.226	0.235
NE × CP			0.064	0.132	0.539	0.274
Starch × CP			0.064	0.171	0.901	0.176
NE			0.230	0.610	0.105	0.296
Starch			0.793	0.101	0.426	0.285
CP			<0.001	<0.001	<0.001	0.049

^ab^Means with different superscripts across the same column are significantly different (*P<* 0.05).

SEM: Standard Error of the Mean.

1 Only three mortalities (*P>* 0.05) were recorded across all treatments at this phase.

2 Net Energy.

3 Crude Protein.

4 Body Weight.

5 Body Weight Gain.

6 Feed Intake.

7 Feed Conversion Ratio.

8 Rapidly Digestible Starch.

9 Slowly Digestible Starch.

**Table 8:** Overall growth performance, cost and age to 2.5 kg body weight of broilers fed diets of varying net energy level, starch source and crude protein level (d 11-35)^1^^,^^2^.

NE^3^	Starch	CP^4^	BWG^5^	FI^6^	FCR^7^	Feed cost per kgBW	Age to 2.5 kg
***NE × Starch × CP***			———g/b——–		g/g	AUD/ kgBW	days
Conventional	RDS^8^	Standard	2433	3822	1.570	0.86	31.38
Conventional	RDS	Reduced	2387	3781	1.584	0.92	32.28
Conventional	SDS^9^	Standard	2493	3876	1.554	0.83	30.74
Conventional	SDS	Reduced	2341	3754	1.604	0.89	32.48
Low	RDS	Standard	2513	3924	1.561	0.83	30.51
Low	RDS	Reduced	2365	3783	1.599	0.93	32.02
Low	SDS	Standard	2506	3872	1.545	0.79	30.59
Low	SDS	Reduced	2346	3762	1.604	0.89	32.40
*SEM*			21.6	33.1	0.0100	0.005	0.218
***NE × Starch***							
Conventional	RDS		2410	3801	1.577	0.89	31.83
Conventional	SDS		2417	3815	1.579	0.86	31.61
Low	RDS		2439	3853	1.580	0.88	31.26
Low	SDS		2426	3817	1.574	0.84	31.50
***NE × CP***							
Conventional		Standard	2463	3849	1.562	0.84^b^	31.06
Conventional		Reduced	2364	3768	1.594	0.91^a^	32.38
Low		Standard	2510	3898	1.553	0.81^c^	30.55
Low		Reduced	2356	3772	1.601	0.91^a^	32.21
***Starch × CP***							
	RDS	Standard	2473	3873	1.566	0.85	30.94
	RDS	Reduced	2376	3782	1.592	0.93	32.15
	SDS	Standard	2500	3874	1.550	0.81	30.67
	SDS	Reduced	2343	3758	1.604	0.89	32.44
***Main Effects***							
***NE***							
Conventional			2414	3808	1.578	0.88	31.72^a^
Low			2433	3835	1.577	0.86	31.38^b^
***Starch***							
RDS			2425	3828	1.579	0.89^a^	31.55
SDS			2422	3816	1.577	0.85^b^	31.56
***CP***							
Standard			2487^a^	3873^a^	1.558^b^	0.83	30.81^b^
Reduced			2360^b^	3770^b^	1.598^a^	0.91	32.30^a^
***P-Values***							
NE × Starch × CP			0.135	0.237	0.595	0.606	0.387
NE × Starch			0.507	0.292	0.599	0.225	0.147
NE × CP			0.080	0.341	0.240	<0.001	0.272
Starch × CP			0.060	0.600	0.055	0.719	0.072
NE			0.220	0.257	0.886	<0.001	0.031
Starch			0.845	0.626	0.781	<0.001	0.957
CP			<0.001	<0.001	<0.001	<0.001	<0.001

^ab^Means with different superscripts across the same column are significantly different (*P<* 0.05).

SEM: Standard Error of the Mean.

1 Only three mortalities (*P>* 0.05) were recorded across all treatments throughout the experimental period.

2 Feed cost per kgBW and age to 2.5 kg were considered from d 0 – 35, while overall growth performance was calculated from d 11 - 35.

3 Net Energy.

4 Crude Protein.

5 Body Weight Gain.

6 Feed Intake.

7 Feed Conversion Ratio.

8 Rapidly Digestible Starch.

9 Slowly Digestible Starch.

There was a two-way interaction between dietary NE and CP levels (*P* *<* 0.001) for feed cost per kg BW (AUD$/kg BW). Feed cost was lower at the standard CP level compared to RCP diets and further decreased when dietary NE was reduced. As a main effect, birds fed SDS had a lower feed cost compared to those fed RDS (0.86 vs. 0.90 AUD/kg BW; *P* *<* 0.001). Birds offered RCP diets took 1.5 more days to reach 2.5 kg BW (32.30 vs 30.81 days, *P* *<* 0.001) compared to those offered standard CP diets.

### Carcass yield

Table 9 presents the carcass yields measured at d 35. A three-way dietary CP × NE × starch source interaction (*P* *=* 0.042) was observed in the absolute leg quarter weight. In birds fed standard CP diets, leg quarter weight was not influenced by dietary NE level or starch source. Two-way dietary NE × CP interactions were observed for absolute weights of breast (*P* *=* 0.011) and tenderloins (*P* *=* 0.042), where the standard CP diets with low NE level led to the highest weights, followed by the standard CP diets with conventional NE level. Both reduced protein diets resulted in markedly lower breast and tenderloin weights, with no statistical difference between the NE levels. A two-way starch × CP interaction was found in absolute (*P* *=* 0.001) and relative (*P* *=* 0.006) weight of breast meat. A greater reduction of the absolute weight due to CP reduction was observed in birds fed SDS (547 to 483 g/b) than in those fed RDS (539 to 512 g/b), whereas the reduction in relative breast weight by CP reduction was only evident in birds fed the SDS diets. Reducing dietary NE by 75 kcal/kg reduced relative fat pads (*P* *<* 0.001) by 9.0%, feeding SDS lowered fat pads (*P* *<* 0.001) by 7.6%, while RCP diets increased the relative fat pads (*P* *<* 0.001) by 19.0%.

**Table 9:** Effect of net energy level, starch source, and dietary crude protein level on absolute and relative carcass weight of broilers on d 35.

NE^1^	Starch	CP^2^	Breast		Tenderloins		Leg Quarters		Empty gizzard		Fat pad	
			Abs. weight^5^, g	Rel. weight^6^, g/kg	Abs. weight^5^, g	Rel. weight^6^, g/kg	Absolute weight^5^, g	Rel. weight^6^, g/kg	Absolute weight^5^, g	Rel. weight^6^, g/kg	Absolute weight^5^, g	Rel. weight^6^, g/kg
***NE × Starch × CP***												
Conventional	RDS^3^	Standard	517	182	102	35.8	584^abc^	206	34.6	12.2	36.3	12.8
Conventional	RDS	Reduced	509	183	90	32.2	554^c^	199	34.2	12.3	41.4	14.9
Conventional	SDS^4^	Standard	535	185	102	35.3	597^ab^	207	35.9	12.4	33.4	11.6
Conventional	SDS	Reduced	480	174	85	30.7	549^c^	199	35.3	12.8	38.6	14.0
Low	RDS	Standard	562	193	105	36.1	604^a^	208	35.8	12.3	32.8	11.3
Low	RDS	Reduced	515	186	87	31.4	564^c^	203	35.4	12.8	38.4	13.8
Low	SDS	Standard	560	193	107	36.8	580^abc^	200	36.3	12.5	30.8	10.6
Low	SDS	Reduced	485	177	86	31.3	566^bc^	206	36.0	13.1	34.4	12.5
*SEM*			7.9	2.5	1.6	0.50	7.5	2.8	0.69	0.26	1.16	0.40
***NE × Starch***												
Conventional	RDS		513	183	96	34.0	569	203	34.4	12.3	38.9	13.9
Conventional	SDS		508	180	93	33.0	573	203	35.6	12.6	36.0	12.8
Low	RDS		538	189	96	33.7	584	205	35.6	12.5	35.6	12.5
Low	SDS		522	185	96	34.0	573	203	36.2	12.8	32.6	11.6
***NE × CP***												
Conventional		Standard	526^b^	184	102^b^	35.6	591	206	35.2	12.3	34.9	12.2
Conventional		Reduced	495^c^	179	87^c^	31.5	552	199	34.7	12.6	40.0	14.5
Low		Standard	561^a^	193	106^a^	36.4	592	204	36.1	12.4	31.8	10.9
Low		Reduced	500^c^	181	86^c^	31.3	565	205	35.7	12.9	36.4	13.2
***Starch × CP***												
	RDS	Standard	539^a^	188^a^	103	36.0	594	207	35.2	12.3	34.5	12.0
	RDS	Reduced	512^b^	185^a^	88	31.8	559	201	34.8	12.5	39.9	14.4
	SDS	Standard	547^a^	189^a^	104	36.0	589	203	36.1	12.5	32.1	11.1
	SDS	Reduced	483^c^	176^b^	85	31.0	557	203	35.6	13.0	36.5	13.3
												
***Main Effects***												
***NE***												
Conventional			510	181^b^	94	33.5	571	203	35.0	12.4	37.4^a^	13.3^a^
Low			530	187^a^	96	33.9	578	204	35.9	12.7	34.1^b^	12.1^b^
***Starch***												
RDS			526	186	96	33.9	576	204	35.0	12.4	37.2^a^	13.2^a^
SDS			515	182	95	33.5	573	203	35.9	12.7	34.3^b^	12.2^b^
***CP***												
Standard			543	188	104	36.0^a^	591	205	35.7	12.4^b^	33.3^b^	11.6^b^
Reduced			497	180	87	31.4^b^	558	202	35.2	12.7^a^	38.2^a^	13.8^a^
***P-Values***												
NE × Starch × CP			0.411	0.651	0.638	0.890	0.042	0.142	0.910	0.872	0.505	0.401
NE × Starch			0.320	0.687	0.280	0.061	0.142	0.510	0.524	0.861	0.965	0.817
NE × CP			0.011	0.075	0.042	0.172	0.289	0.053	0.904	0.455	0.722	0.927
Starch × CP			0.001	0.006	0.069	0.201	0.695	0.221	0.927	0.582	0.550	0.768
NE			<0.001	0.002	0.132	0.296	0.179	0.489	0.067	0.170	<0.001	<0.001
Starch			0.062	0.037	0.355	0.282	0.524	0.261	0.089	0.090	<0.001	<0.001
CP			<0.001	<0.001	<0.001	<0.001	<0.001	0.129	0.370	0.041	<0.001	<0.001

^abc^Means with different superscripts across the same column are significantly different (*P<* 0.05).

SEM: Standard Error of the Mean.

1 Net Energy.

2 Crude Protein.

3 Rapidly Digestible Starch.

4 SlowlyDigestible Starch.

5 Absolute weight.

6 Relative weight.

### Apparent Jejunal and Ileal nutrient digestibility

The apparent nutrient digestibility of dry matter, gross energy, CP and starch in the distal jejunum and ileum is summarized in Table 10. Reducing dietary NE reduced the apparent digestibility of DM (*P* *=* 0.001), gross energy (*P* *=* 0.023) and protein (*P* *=* 0.020) in the distal jejunum compared to conventional NE diets. The ileal gross energy digestibility was also lower (*P* *=* 0.032) in birds fed low NE diets compared to those fed conventional NE diets. Compared to feeding RDS, birds fed SDS presented lower (*P* *<* 0.05) apparent digestibility of DM, energy and starch in both distal jejunum and ileum. Reducing CP improved apparent jejunal protein (N) digestibility compared to conventional CP diets (*P* *=* 0.006). A two-way interaction between NE and starch source (*P* *=* 0.009) was observed for distal ileal protein digestibility coefficient, where feeding RDS improved the digestibility in conventional NE diets but not in the low NE diets. There was also an interaction between dietary NE and CP levels in ileal CP digestibility (*P* *=* 0.021), where birds fed RCP diets showed markedly lower digestibility only with the conventional NE diet.

**Table 10:** Apparent nutrient digestibility of broilers fed diets of varying net energy level, starch source and crude protein level.

NE^1^	Starch	CP^2^	Jejunal digestibility coefficient				Ileal digestibility coefficient			
			DM^3^	Energy	Protein	Starch	DM	Energy	Protein	Starch
***NE × Starch × CP***										
Conventional	RDS^4^	Standard	0.656	0.634	0.746	0.934	0.754	0.782	0.860	0.963
Conventional	RDS	Reduced	0.651	0.693	0.736	0.937	0.735	0.762	0.828	0.965
Conventional	SDS^5^	Standard	0.616	0.631	0.690	0.900	0.716	0.747	0.826	0.944
Conventional	SDS	Reduced	0.630	0.654	0.720	0.901	0.730	0.753	0.806	0.958
Low	RDS	Standard	0.614	0.633	0.662	0.931	0.727	0.753	0.830	0.971
Low	RDS	Reduced	0.632	0.646	0.697	0.926	0.731	0.753	0.827	0.964
Low	SDS	Standard	0.582	0.622	0.687	0.896	0.706	0.735	0.828	0.955
Low	SDS	Reduced	0.600	0.629	0.697	0.890	0.726	0.745	0.828	0.953
*SEM*			0.0105	0.0125	0.0219	0.0060	0.0091	0.0094	0.0072	0.0053
***NE × Starch***										
Conventional	RDS		0.653	0.664	0.741	0.935	0.744	0.772	0.844^a^	0.964
Conventional	SDS		0.623	0.642	0.705	0.901	0.723	0.750	0.816^b^	0.951
Low	RDS		0.623	0.640	0.679	0.928	0.729	0.753	0.828^ab^	0.968
Low	SDS		0.591	0.625	0.692	0.893	0.716	0.740	0.828^ab^	0.954
***NE × CP***										
Conventional		Standard	0.636	0.633	0.718	0.917	0.735	0.765	0.843^a^	0.954
Conventional		Reduced	0.640	0.674	0.728	0.919	0.733	0.758	0.817^b^	0.961
Low		Standard	0.598	0.627	0.674	0.914	0.717	0.744	0.829^ab^	0.963
Low		Reduced	0.616	0.637	0.697	0.908	0.728	0.749	0.828^ab^	0.959
***Starch × CP***										
	RDS	Standard	0.635	0.634	0.704	0.933	0.741	0.767	0.845	0.967
	RDS	Reduced	0.641	0.670	0.717	0.931	0.733	0.757	0.827	0.965
	SDS	Standard	0.599	0.626	0.688	0.898	0.711	0.741	0.827	0.950
	SDS	Reduced	0.615	0.641	0.708	0.896	0.728	0.749	0.817	0.955
										
***Main Effects***										
***NE***										
Conventional			0.638^a^	0.653^a^	0.723^a^	0.918	0.734	0.761^a^	0.830	0.958
Low			0.607^b^	0.632^b^	0.686^b^	0.911	0.723	0.746^b^	0.828	0.961
***Starch***										
RDS			0.638^a^	0.652^a^	0.710	0.932^a^	0.737^a^	0.762^a^	0.836	0.966^a^
SDS			0.607^b^	0.634^b^	0.698	0.897^b^	0.719^b^	0.745^b^	0.822	0.953^b^
***CP***										
Standard			0.617	0.630^b^	0.696	0.916	0.726	0.754	0.836	0.958
Reduced			0.628	0.656^a^	0.712	0.913	0.730	0.753	0.822	0.960
***P-Values***										
NE × Starch × CP			0.518	0.407	0.308	0.914	0.513	0.570	0.704	0.656
NE × Starch			0.917	0.706	0.125	0.962	0.513	0.519	0.009	0.972
NE × CP			0.365	0.086	0.700	0.379	0.302	0.370	0.021	0.131
Starch × CP			0.530	0.240	0.822	0.874	0.062	0.179	0.439	0.305
NE			0.001	0.023	0.020	0.101	0.096	0.032	0.701	0.420
Starch			0.001	0.048	0.449	<0.001	0.011	0.014	0.008	0.001
CP			0.128	0.006	0.295	0.616	0.484	0.910	0.010	0.658

^ab^Means with different superscripts across the same column are significantly (*P<* 0.05) different.

SEM: Standard Error of the Mean.

1 Dietary Net Energy Level.

2 Crude Protein.

3 Dry Matter.

4 Rapidly Digestible Starch.

5 Slowly Digestible Starch.

### Jejunal and Ileal nutrient disappearance rates

Table 11 shows the CP and starch disappearance rates and the starch:protein disappearance rate ratios (**S:P**) in the distal jejunum and ileum. A two-way NE and starch interaction was observed on the jejunal and ileal protein and starch disappearance rates. Jejunal protein disappearance rate was higher in RDS diets compared to SDS diets when diets containing conventional NE, but it did not differ among starch sources in low NE diets. However, the ileal protein disappearance rate was lowest (*P* *<* 0.001) for SDS with conventional NE. Similarly, the jejunal and ileal protein and starch disappearance rates were impacted (*P* *<* 0.05) by the two-way NE × CP interactions. Reducing dietary CP lowered protein disappearance rate with conventional and low NE levels in the distal jejunum (*P* *=* 0.027) and ileum (*P* *<* 0.001), although the gap between the standard and RCP was much higher under conventional NE compared to low NE. The birds’ starch disappearance rate was significantly affected (*P* *<* 0.005) by NE × starch interaction in the distal jejunum and ileum. Feeding SDS lowered jejunal starch disappearance rate (*P* *=* 0.050) compared to RDS in birds fed conventional NE diets, but not in those fed low NE diets. The starch × CP interaction also significantly affected starch disappearance rate (*P* *<* 0.005) in the distal jejunum and ileum. Birds fed conventional NE diets showed a lower ileal starch disappearance rate compared to those fed low NE diets when SDS was used, but not with RDS.

**Table 11:** Nutrient disappearance rates and starch:protein disappearance rate ratio of broilers fed diets of varying net energy level, starch source and crude protein level.

NE^1^	Starch	CP^2^	Jejunal nutrient disappearance rate, g/b/d			Ileal nutrient disappearance rate, g/b/d		
			Protein	Starch	J-S:P^3^	Protein	Starch	I-S:P^4^
***NE × Starch × CP***								
Conventional	RDS^5^	Standard	28.84	88.94	3.10	33.13	91.31	2.76
Conventional	RDS	Reduced	22.89	92.17	4.02	25.80	95.08	3.69
Conventional	SDS^6^	Standard	25.28	81.24	3.26	30.31	85.20	2.81
Conventional	SDS	Reduced	20.96	88.79	4.24	23.58	94.83	4.02
Low	RDS	Standard	24.10	89.22	3.75	30.30	92.98	3.07
Low	RDS	Reduced	22.19	89.32	4.04	26.39	93.04	3.52
Low	SDS	Standard	26.04	85.75	3.30	31.42	91.45	2.91
Low	SDS	Reduced	22.45	90.48	4.04	26.69	96.86	3.63
*SEM*			0.734	1.537	0.133	0.429	1.454	0.026
***NE × Starch***								
Conventional	RDS		25.87^a^	90.56^a^	3.56^b^	29.46^a^	93.20^ab^	3.22^c^
Conventional	SDS		23.12^b^	85.01^b^	3.75^ab^	26.95^b^	90.02^b^	3.42^a^
Low	RDS		23.15^b^	89.27^a^	3.89^a^	28.35^a^	93.01^ab^	3.30^b^
Low	SDS		24.24^ab^	88.12^ab^	3.67^ab^	29.06^a^	94.15^a^	3.27^bc^
***NE × CP***								
Conventional		Standard	27.06^a^	85.09	3.18^c^	31.72^a^	88.26	2.78^d^
Conventional		Reduced	21.93^c^	90.48	4.13^a^	24.69^c^	94.96	3.85^a^
Low		Standard	25.07^b^	87.48	3.52^b^	30.86^a^	92.21	2.99^c^
Low		Reduced	22.32^c^	89.90	4.04^a^	26.54^b^	94.95	3.58^b^
***Starch × CP***								
	RDS	Standard	26.47	89.08^a^	3.42	31.71	92.14^ab^	2.91^c^
	RDS	Reduced	22.54	90.74^a^	4.03	26.10	94.06^a^	3.61^b^
	SDS	Standard	25.66	83.49^b^	3.28	30.87	88.33^b^	2.86^c^
	SDS	Reduced	21.70	89.64^a^	4.14	25.13	95.84^a^	3.83^a^
***Main Effects***								
***NE***								
Conventional			24.49	87.78	3.66	28.20	91.61	3.32
Low			23.70	88.69	3.78	28.70	93.58	3.28
***Starch***								
RDS			24.51	89.91	3.73	28.91	93.10	3.26
SDS			23.68	86.57	3.71	28.00	92.09	3.34
***CP***								
Standard			26.06	86.29	3.35	31.29	90.23	2.89
Reduced			22.12	90.19	4.09	25.62	94.95	3.72
***P-Values***								
NE × Starch × CP			0.118	0.945	0.283	0.249	0.901	0.802
NE × Starch			0.001	0.050	0.034	<0.001	0.041	<0.001
NE × CP			0.027	0.179	0.027	<0.001	0.060	<0.001
Starch × CP			0.980	0.046	0.189	0.852	0.009	<0.001
NE			0.133	0.409	0.189	0.107	0.061	0.054
Starch			0.120	0.004	0.861	0.005	0.328	<0.001
CP			<0.001	0.001	<0.001	<0.001	<0.001	<0.001

^abc^Means with different superscripts across the same column are significantly (*P<* 0.05) different.

SEM: Standard Error of the Mean.

1 Dietary Net Energy Level.

2 Crude Protein.

3 Starch:Protein Disappearance Rate Ratio in the jejunum.

4 Starch:Protein Disappearance Rate Ratio in the ileum.

5 Rapidly Digestible Starch.

6 Slowly Digestible Starch.

The interaction between NE and starch significantly influenced S:P ratios in both the distal jejunum and ileum of broilers. Feeding RDS resulted in lower S:P ratios in the jejunum for conventional NE than for low NE (3.56 vs 3.89, *P* *=* 0.034), whereas SDS produced similar S:P ratios at both NE levels. However, in the ileum, S:P was significantly higher (*P* *<* 0.001) for SDS than RDS when fed conventional NE diets. The NE × CP interaction affected (*P* *<* 0.05) S:P ratios in broilers. Reducing dietary CP increased S:P ratios for both conventional and low NE levels in the jejunum (*P* *=* 0.027) and ileum (*P* *<* 0.001). The two-way interaction between starch and CP significantly impacted ileal S:P ratios (*P* *<* 0.001), as decreasing dietary CP raised ileal S:P ratios when fed either RDS or SDS.

### Apparent metabolizable energy, efficiency of energy utilization and nitrogen retention

The apparent metabolizable energy, efficiency of gross energy utilization and nitrogen retention are presented in Table 12. A three-way interaction among NE, starch source and CP level was observed for AME (*P* *=* 0.020) and AMEn (*P* *=* 0.017) in broilers. SDS affected AME only under low-NE conditions. RDS maintained similar AME across low- and conventional-NE diets, regardless of CP reduction. Conventional-NE diets yielded similar AMEn values, irrespective of starch digestion rate or dietary CP level. However, in low-NE diets, AMEn was reduced, except in birds offered RDS-SCP diets. A two-way NE × CP interaction (*P* *=* 0.019) was observed for AME:GE, in which CP reduction increased the ratio only in birds fed conventional NE diets but not in those fed low NE diets. The interaction between starch and CP affected the birds’ N retention (*P* *=* 0.047). RCP diets consistently yielded higher N retention, irrespective of starch source.

**Table 12:** Apparent metabolizable energy and nitrogen retention by broiler chickens fed diets of varying net energy level, starch source, and dietary crude protein levels.

NE^1^	Starch	CP^2^	AME^3^	AMEn^4^	AME:GE	N retention, %
***NE × Starch × CP***						
Conventional	RDS^5^	Standard	3059^ab^	2821^a^	0.781	65.49
Conventional	RDS	Reduced	3096^a^	2785^a^	0.801	73.55
Conventional	SDS^6^	Standard	3058^abc^	2788^a^	0.769	64.85
Conventional	SDS	Reduced	3062^ab^	2763^a^	0.791	69.55
Low	RDS	Standard	3061^ab^	2798^a^	0.781	65.23
Low	RDS	Reduced	3027^bc^	2699^b^	0.782	72.40
Low	SDS	Standard	2956^d^	2677^b^	0.760	67.81
Low	SDS	Reduced	2991^cd^	2678^b^	0.775	70.90
SEM			14.9	12.6	0.0038	1.370
***NE × Starch***						
Conventional	RDS		3077	2803	0.791	69.52
Conventional	SDS		3060	2776	0.780	67.20
Low	RDS		3044	2749	0.782	68.82
Low	SDS		2974	2678	0.767	69.36
***NE × CP***						
Conventional		Standard	3058	2805	0.775^b^	65.17
Conventional		Reduced	3079	2774	0.796^a^	71.55
Low		Standard	3009	2738	0.771^b^	66.52
Low		Reduced	3009	2689	0.779^b^	71.65
***Starch × CP***						
	RDS	Standard	3060	2809	0.781	65.36
	RDS	Reduced	3061	2742	0.792	72.97
	SDS	Standard	3007	2732	0.765	66.33
	SDS	Reduced	3026	2720	0.783	70.23
***Main Effects***						
***NE***						
Conventional			3069	2789	0.785	68.36
Low			3009	2713	0.775	69.09
***Starch***						
RDS			3061	2776	0.786^a^	69.17
SDS			3017	2727	0.774^b^	68.28
***CP***						
Standard			3033	2771	0.773	65.85^b^
Reduced			3044	2731	0.787	71.60^a^
***P-Values***						
NE × Starch × CP			0.020	0.017	0.314	0.854
NE × Starch			0.017	0.019	0.472	0.147
NE × CP			0.336	0.317	0.019	0.522
Starch × CP			0.406	0.003	0.150	0.061
NE			<0.001	<0.001	<0.001	0.456
Starch			<0.001	<0.001	<0.001	0.365
CP			0.325	<0.001	<0.001	<0.001

^a-d^Means with different superscripts across the same column are significantly (*P<* 0.05) different.

SEM: Standard Error of the Mean.

1 Dietary Net Energy Level.

2 Crude Protein.

3 Apparent metabolizable energy.

4 Apparent metabolizable energy corrected for Nitrogen retention.

5 Rapidly Digestible Starch.

6 Slowly Digestible Starch.

### Correlations between performance parameter and digestibilities

To eliminate the influence of feed intake, disappearance rate ratios were used in the correlation analyses. Abdominal fat pad weight was positively correlated with the ileal starch-to-protein disappearance rate ratio (r = 0.610; P < 0.001; Fig. 1). Breast weight was positively correlated with ileal CP disappearance rate (r = 0.465; *P* *<* 0.001) but negatively with ileal starch: protein disappearance rate ratio (r = – 0.485; *P* *<* 0.001)

**Fig. 1:**
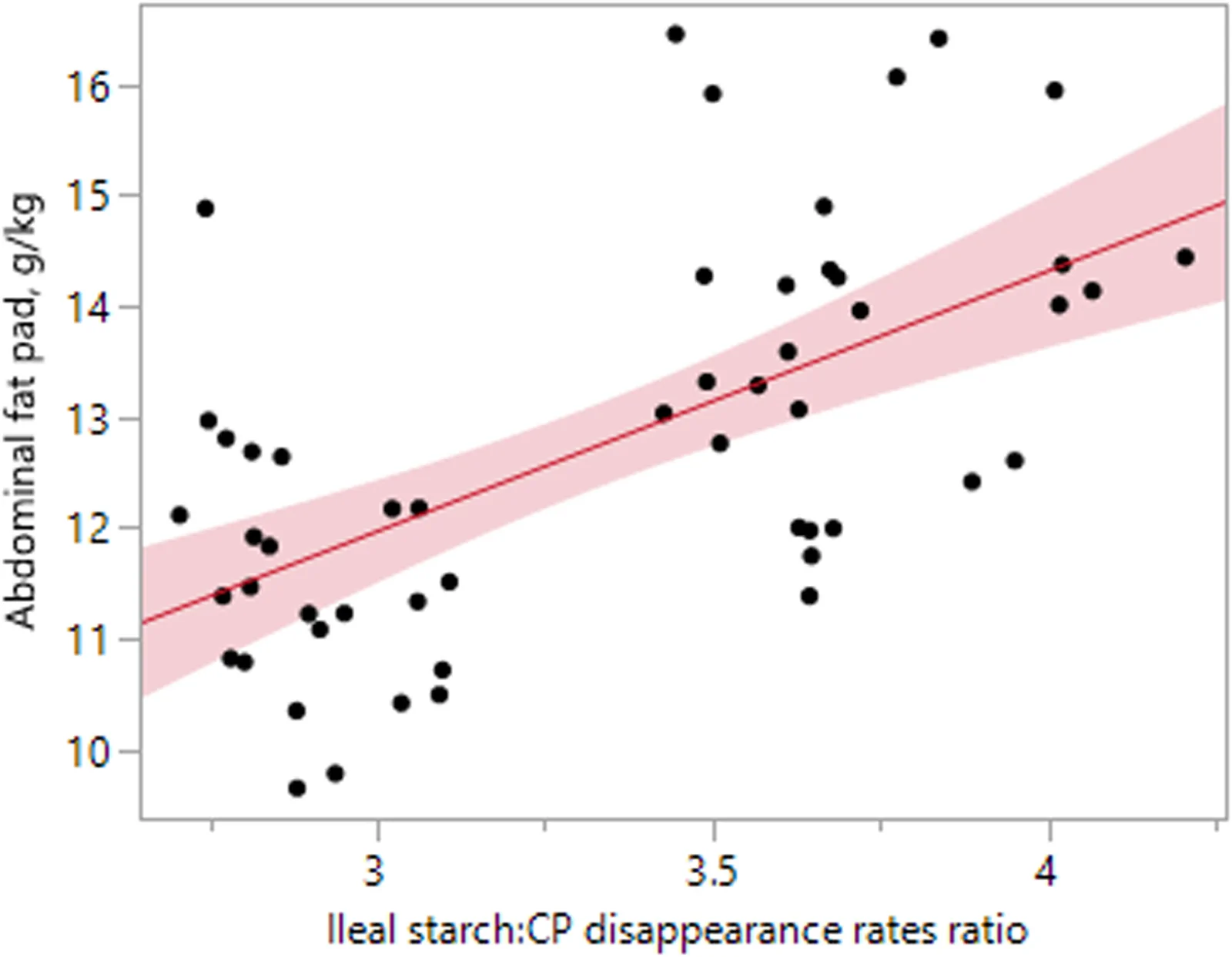
Relationship between abdominal fat pad and ileal starch:CP disappearance rates ratio in broilers [*y*(fat pad, g/kg) = 4.933 + 2.346 * ileal starch:CP disappearance rates ratio; r = 0.610; R^2^ = 0.372; *P<* 0.001; *n* = 54].

## Discussion

In the present study, a 3 percentage point reduction in dietary CP reduced feed intake by 2.6% and weight gain by 4.8%, while increasing FCR by 3.5 points (*P* *<* 0.05). This result is somewhat inconsistent with the findings of Chrystal et al. (2021b), who reported that a 3 percentage point reduction in dietary CP did not compromise growth performance in corn-based diets. In both experiments, a similar set of amino acid requirements was considered in feed formulation, including lysine, total sulfur amino acids, threonine, branched-chain amino acids, tryptophan, arginine, glycine equivalent, and histidine; however, the dietary crude protein levels were quite different between the two studies, which could have contributed to the differences observed. In the Chrystal et al. (2021b) study, dietary CP was reduced from 220 to 190 g/kg for broiler chickens from 14 to 35 days of age, whereas in the present study, dietary CP was reduced from 205 to 175 g/kg during the grower phase (11 to 25 days) and from 190 to 160 g/kg during the finisher phase (26 to 35 days). The RCP diets in the present study had substantially lower dietary crude protein concentrations, which may have led to deficiencies in non-essential amino acids that are not typically considered in standard feed formulation. These include phenylalanine, tyrosine, proline, alanine, aspartic acid, and glutamic acid.

The feed intake was markedly reduced in birds offered RCP diets in the present study. Wheat was included at 200 g/kg in the diets to ensure adequate pellet quality. Indeed, there was no correlation between pellet durability index and feed intake in the present study. Despite the interaction between dietary net energy and crude protein on pellet durability index (Table 5), the RCP diets generally had a higher pellet durability index in the present study, which was somewhat unexpected. However, this may have been due to the increased inclusion of cereal grains (corn) and the reduced inclusion of canola oil in the RCP diets. The inclusion of field peas did not substantially alter NBAA inclusion. For example, in the SCP diets, the inclusion of field peas changed NBAA inclusion from 5.57 to 6.91 g/kg in the conventional NE diets, whereas in the RCP diets, field pea inclusion changed NBAA inclusion from 24.67 to 25.39 g/kg. In addition, added oil was maintained at a constant level across diets containing the same CP. Therefore, the observed change in pellet quality is most likely attributable to the starch functionality of field peas *per se*.

It is worth noting that the reduction in NE was achieved differently in the SCP and RCP diets. In the SCP diets, NE reduction was achieved by reducing the inclusion of added fat (canola oil), whereas in the RCP diets, NE reduction was achieved by including millrun. However, no CP × NE interaction was observed for growth performance. In contrast, a significant interaction was detected for feed cost per kilogram of body weight gain. Specifically, reducing NE in the SCP diets decreased feed cost from AUD $0.84 to AUD $0.81 per kilogram of body weight gain, whereas reducing NE had no effect on production costs in the RCP diets. It should be noted that this comparison is based on ingredient prices at the time of the study, when oil prices were relatively high; therefore, the economic outcomes may differ under different market conditions and ingredient price structures.

The improvement in FCR observed when dietary NE was reduced by 75 kcal/kg during the grower phase (d 11–25), but not during the finisher phase (d 25–35), may suggest an age-dependent performance response to dietary energy concentration. Caldas et al. (2019) reported that younger broilers prioritize lean tissue accretion, whereas maintenance requirements and lipid deposition become increasingly important with advancing age. Thus, the moderate reduction in NE during the grower phase may have resulted in a more favorable balance between energy supply and the nutrients required for protein accretion, thereby improving feed efficiency. Inclusion of peas as a strategy to slow starch digestion reduced FCR in broilers during the grower phase (d 11-25) by 1.8 points (1.514 vs. 1.532; *P* = 0.003). Sydenham et al. (2017) suggested that a lower starch-protein disappearance rate ratio may be beneficial for broiler performance and they reported positive correlations between the starch-protein disappearance rate ratio in the proximal jejunum and both weight gain (*r* = 0.849; *P* < 0.001) and FCR (*r* = 0.838; *P* < 0.001). Consistent with these findings, positive relationships were observed between the starch to protein disappearance rate ratio and finisher FCR in the distal jejunum (*r* = 0.390, *P* = 0.004) and ileum (*r* = 0.503, *P* < 0.001) in the present study. Furthermore, we found relative breast weight to positively correlate with ileal CP disappearance rate (r = 0.465; *P* *<* 0.001) but negatively with ileal starch:CP disappearance rate ratio (r = – 0.485; *P* *<* 0.001). As SDS diets support lower ileal CP disappearance rate (Table 11), this could possibly reduce protein accretion, especially when dietary CP is limiting, as seen in this study. However, FCR was positively correlated with ileal (r = 0.487, *P* *=* 0.0002) and jejunal (r = 0.450, *P* *<* 0.001) starch disappearance rates. This may suggest a potential positive impact of slow starch digestion or lower glucose uptake on broilers’ performance.

Increased fat deposition has been a major challenge in RCP diets. Efforts to reduce fat deposition in broilers offered RCP diets by lowering dietary ME (Kamran et al., 2008; Macelline et al., 2023; Strifler et al., 2023) were unsuccessful. However, Mahmood et al. (2024) found that reducing dietary starch:protein ratio or including pea in RCP diets lowered blood triglycerides, which has been associated with fat deposition (Huang et al., 2015; Zou et al., 2020). In the present study, including field peas as an intended SDS source lowered relative fat pad weights, while reducing dietary CP increased relative fat pad weights by 19.0%. SDS inclusion reduced both the extent and the rate of starch digestion, as reflected by decreased starch digestibility in the jejunum and ileum. Consequently, it is difficult to determine whether the observed reduction in fat pad deposition resulted from a slower rate of glucose release or from a reduction in total starch digestion. Notably, abdominal fat pad weight was positively correlated with ileal starch disappearance rate (r = 0.404; *P* *=* 0.003) and the ileal starch:CP disappearance rate ratio (r = 0.610; *P* *<* 0.001) but negatively correlated with ileal CP disappearance rate (r = − 0.515; *P* *<* 0.001) in the present study (Figure 1).

As expected, NE reduction decreased energy digestibility in both the jejunum and ileum; however, this did not compromise growth performance. In fact, a reduction of 75 kcal/kg NE was well tolerated in the present study, regardless of dietary CP level or starch source, with no adverse effects on growth performance throughout all growth phases. Moreover, reducing dietary NE by 75 kcal/kg reduced relative fat pad weight by 9%. Modern broilers appear to be more sensitive to dietary protein and amino acid supply than to moderate reductions in dietary energy, and numerous studies suggested dietary energy can be further reduced from breeder recommendations without compromising growth performance (Toghyani et al., 2025). Consistent with this, Rosa et al. (2007) had earlier found that lowering dietary energy (by 250 kcal/kg ME) reduced abdominal fat by approx. 27%. Similarly, a more recent study by Toghyani et al. (2025) showed that reducing dietary AME by 150 kcal/kg reduced fat pad by around 16%. The present study is perhaps the first to evaluate the impact of NE reduction in the context of reduced-CP diets. The findings demonstrate that NE-based formulation, which allows greater incorporation of fiber-rich by-product ingredients, has the potential to reduce feed costs, maintain growth performance, and decrease fat pad deposition in broilers fed reduced-CP diets.

The current study showed a higher gizzard relative weight for the RCP diets, which is different from expectation. Again, this could be associated with better pellet quality, as RCP diets had higher PDI, potentially due to more cereal grain and less oil inclusions. As expected, regardless of dietary NE level or field pea inclusion, RCP diets resulted in greater nitrogen retention than SCP diets. This highlights the confirmed benefit of RCP diets beyond reducing SBM inclusion, improving overall nitrogen retention and reducing nitrogen excretion (Chrystal et al., 2020). Studies have previously linked lower starch digestibility to lower AME in broilers (Svihus, 2006; Talibov, 2017). The present study is consistent with these findings, where SDS is shown to produce lower starch digestibility than RDS (Table 10), and NE-SDS diets led to lower analyzed AME.

## Conclusions

Reducing dietary CP by 3 percentage points from 205 to 175 g/kg in grower diets and from 190 to 160 g/kg in finisher diets compromised growth performance, despite supplementation with key essential amino acids, and this suggests that limitations in non-essential amino acid supply may constrain the application of reduced-CP diets. In contrast, reducing dietary NE by 75 kcal/kg did not adversely affect overall performance, while decreasing abdominal fat deposition. Inclusion of field peas as a source of slowly digestible starch improved grower-phase FCR and reduced fat pad weight, although these responses were not maintained during the finisher phase. Collectively, these findings demonstrate that moderate reductions in dietary NE can be successfully incorporated into reduced-CP diets to reduce fat pad deposition. However, this finding should be verified in RCP diets where growth performance is not compromised.

## Author Contribution

Damilola U. Kareem led the experimental work, data collection and analysis, and prepared the first draft of the manuscript. Eunjoo Kim and Shemil P. Macelline contributed to the study design, experimental work, data analysis, supervision of the project, and manuscript revision. Tahir Mahmood and Yves Mercier contributed to the study conception and design, provided methodological input, assisted with securing project funding, data interpretation and manuscript revision. Mehdi Toghyani formulated the experimental diets, contributed to data interpretation and manuscript revision. Peter H. Selle contributed to the study conception, interpretation of the findings and reviewed the manuscript. Sonia Y. Liu conceived and supervised the project, secured funding, contributed to the study design and data interpretation, and reviewed and revised the manuscript. All authors read and approved the final manuscript.

## Disclosures

The authors declare no financial or personal relationships that could inappropriately influence our work, and there is no professional or other personal interest of any nature or kind in any product, service and/or company that could be construed as influencing the content of this paper. Although, the fourth and fifth authors, Mahmood Tahir and Yves Mercier, are employees of the funding body of this research, Adisseo France S.A.S., France, their involvements were transparent and does not compromise the integrity of the findings.
